# The ERβ5 splice variant increases oestrogen responsiveness of ERα^pos^ Ishikawa cells

**DOI:** 10.1530/ERC-19-0291

**Published:** 2019-11-27

**Authors:** Frances Collins, Nozomi Itani, Arantza Esnal-Zufiaurre, Douglas A Gibson, Carol Fitzgerald, Philippa T K Saunders

**Affiliations:** 1 The University of Edinburgh Centre for Inflammation Research, Queen’s Medical Research Institute, Edinburgh, UK

**Keywords:** estrogen receptor, endometrium, estrogen, carcinoma

## Abstract

Endometrial cancer is a common gynaeological malignancy: life time exposure to oestrogen is a key risk factor. Oestrogen action is mediated by receptors encoded by *ESR1* (ERα) and *ESR2* (ERβ): ERα plays a key role in regulating endometrial cell proliferation. A truncated splice variant isoform (ERβ5) encoded by *ESR2* is highly expressed in cancers. This study explored whether ERβ5 alters oestrogen responsiveness of endometrial epithelial cells. Immunhistochemistry profiling of human endometrial cancer tissue biopsies identified epithelial cells co-expressing ERβ5 and ERα in stage I endometrial adenocarcinomas and post menopausal endometrium. Induced co-expression of ERβ5 in ERα^pos^ endometrial cancer cells (Ishikawa) significantly increased ligand-dependent activation of an ERE-luciferase reporter stimulated by either E2 or the ERα-selective agonist 1,3,5-(4-hydroxyphenyl)-4-propyl-1H-pyrazole (PPT) compared to untransfected cells. Fluorescence recovery after photobleaching (FRAP) analysis of tagged yellow fluorescent protein (YFP)-ERβ5 transfected into Ishikawa cells revealed that incubation with E2 induced a transient reduction in intra-nuclear mobility characterised by punctate protein redistribution which phenocopied the behaviour of ERα following ligand activation with E2. In ERα^neg^ MDA-MD-231 breast cancer cells, there was no E2-dependent change in mobility of YFP-ERβ5 and no activation of the ERE reporter in cells expressing ERβ5. In conclusion, we demonstrate that ERβ5 can act as heterodimeric partner to ERα in Ishikawa cells and increases their sensitivity to E2. We speculate that expression of ERβ5 in endometrial epithelial cells may increase the risk of malignant transformation and suggest that immunostaining for ERβ5 should be included in diagnostic assessment of women with early grade cancers.

## Introduction

Endometrial cancer is the most common gynaecological malignancy in the developed world with the majority presenting as abnormal bleeding in post-menopausal women; the incidence of this cancer is increasing in parallel with changing demographics characterized by an aging population and increased prevalence of obesity (Sanderson *et al.* 2017). Clinically, endometrial cancers are routinely classified as having a type I or type II phenotype, with the former being oestrogen dependent and the latter oestrogen independent (Bokhman 1983).

A study examining the risk factors for type I and type II endometrial cancers based on 14,069 cancer cases, reported that risk of developing either type of malignancy was influenced by parity, oral contraceptive use, age at menarche, and diabetes but higher BMI had a greater effect on the risk of developing a type I tumour (Setiawan *et al.* 2013). A genome wide significant association between endometrial cancer and a *CYP19A1* (aromatase gene) SNP associated with increased circulating E2 concentrations has been reported (Thompson *et al.* 2016). In pre-menopausal women the primary source of endogenous oestrogens are the ovaries although local biosynthesis can also occur in the endometrium (Gibson & Saunders 2012, Gibson *et al.* 2013). After menopause synthesis of oestrogens in non-ovarian sites such as adipose tissue predominates but expression of oestrogen biosynthetic enzymes including CYP19A1, HSD17B1 and sulphatase within endometrial cancer tissues is consistent with intracrine biosynthesis of bioactive oestrogens from blood-borne steroid precursors. For example sulphatase converts of E1-S to E1, and HSD17B1 can convert E1 to E2 (reviewed in Rizner *et al.* 2017, Sinreih *et al.* 2017).

Oestrogenic ligands (endogenous or synthetic) can induce phenotypic changes that can contribute to increased cancer risk including proliferation, angiogenesis, migration and epithelial-to-mesenchymal transition by binding to oestrogen receptors which act as ligand-activated transcription factors. In women the key nuclear oestrogen receptors are ERα, encoded by *ESR1*, and ERβ encoded by *ESR2*: both receptors are expressed in endometrial tissue during the normal menstrual cycle (Critchley *et al.* 2002). Studies using knockout mice have highlighted the importance of *Esr1* in mediating the proliferative effects of oestrogens on endometrial epithelial cells (Winuthayanon *et al.* 2017). A study of ~6000 cancer patients reported a strong risk signal for endometrioid cancers was located in a promoter of *ESR1* (O’Mara *et al.* 2015).

In common with other members of the nuclear receptor family (van der Vaart & Schaaf 2009), the *ESR1* and *ESR2* genes are subject to alternative splicing with both C terminal and exon-skipping isoforms identified in cancer cell lines and human tissues including the testis (Saunders *et al.* 2002). In this paper we have focused on a C-terminal splice variant of *ESR2* called ERβ5 which contains an identical sequence encoded by exons 1–7 of the WT protein (sometimes called ERβ1 to distinguish it from variant isoforms) but incorporates a unique 8th exon. The resultant protein has an intact DNA-binding domain but lacks amino acids in the E/F domains of ERβ1 which contribute to the ligand-binding pocket and binding of co-factors critical for a robust response to ligand (Poola *et al.* 2005, Gibson & Saunders 2012). This splice variant does not exist in rodents. We have previously developed a specific antibody to the unique C-terminus of the protein and confirmed expression in endometrial and other cancers (Wong *et al.* 2005, Shaaban *et al.* 2008, Collins *et al.* 2009). Despite lacking an intact ligand-binding domain, cell line studies have reported that co-expression of ERβ5 can alter transcriptional activity of ERs in response to oestrogens. For example, in COS7 cells (SV40 transformed monkey kidney cells) ERβ5 was able to bind DNA in a gel shift assay and inhibited the activity of ERα, but not ERβ1, on a TGF-beta3-CAT gene reporter (Peng *et al.* 2003, Poola *et al*. 2005). In HEK293 (embryonic kidney) cells ERβ1:ERβ5 heterodimers induced greater expression of an ERE reporter gene in response to incubation with E2 but ERα co-transfection was not tested (Leung *et al.* 2006). Overexpression of ERβ5 in PC3 cells (metastatic, ERβ^pos^, prostate cancer cells) increased cell migration (Leung *et al.* 2010). Taken together these results suggest that expression of ERβ5 can have an impact on oestrogen responsiveness and therefore has the potential to alter oestrogen-driven progression of malignancy in cancers, albeit in a cell context-dependent manner.

In support of this, some reports suggest immunoexpression of ERβ5 could be a useful prognostic indicator in cancer. Wimberley *et al*. (2014) reported immunoexpression of ERβ5 was associated with worse outcome in triple-negative/HER-2 breast cancer patients. In a study on prostate cancer, cytoplasmic ERβ5 staining was associated with a reduced survival time to post-operative metastases (Leung *et al*. 2010). Over-expression of ERβ5 has also been reported in colon cancers (Wong *et al*. 2005), glioma (Li *et al.* 2013), cancers of the ovary (Ciucci *et al.* 2014) and of the thymus (Li *et al.* 2015); however, to date, the impact of ERβ5 in endometrial cancers is unknown.

In this study we have demonstrated co-expression with of ERβ5 with ERα in epithelial cell nuclei of stage I endometrial adenocarcinomas and provided novel evidence to support formation of ERα:ERβ5 heterodimers in cell line model of endometrial adenocarcinoma (Ishikawa). These results suggest the presence of ERβ5 in ERα-positive cells may augment the oestrogen sensitivity of cells and drive malignant transformation.

## Materials and methods

### Patients and tissue collection

Endometrial adenocarcinomas had previously been recovered from post-menopausal women (*n* = 101) undergoing total abdominal hysterectomy. Written informed consent was obtained from all patients and ethical approval granted by the Lothian Research Ethics committee (LRE 1999/6/4) as detailed in Collins *et al*. (2009). Additional (control) samples (*n* = 9) were obtained from women who were postmenopausal (14 months to 26 years after their self-reported last menstrual period) and attending clinics for treatment of benign gynaecological conditions, including heavy menstrual bleeding. In all cases women were recruited by dedicated research nurses and written consent was obtained prior to tissue collection under Research Ethics 10/S1402/59 or 07/S1103/29. Tissue for immunohistochemistry was fixed in 4% neutral buffered formalin overnight at 4°C. Tissue for RNA extraction was collected in RNALater (Qiagen). All cancers were confined to the uterus (stage I). Grading of tissues as well (G1), moderately (G2) or poorly differentiated (G3), was performed by an expert gynaecological pathologist according to the FIGO (International Federation of Obstetrics and Gynaecology) grading system (Scully *et al.* 1994). We have previously used a subset of samples from this tissue archive and conducted DAB immunohistochemistry to investigate immunoexpression of individual *ESR2*-encoded proteins (Collins *et al*. 2009).

### Cell lines

Endometrial epithelial adenocarcinoma Ishikawa cells were originally derived from a well-differentiated adenocarcinoma in a 39-year-old pre-menopausal woman (Nishida *et al.* 1985): catalogue no 99040201 (ECACC, Wiltshire, UK). RL95-2 endometrial epithelial carcinoma cells were derived from a moderately differentiated 64 year old, catalogue no RL95-2 ATCC-CRL-1671 (LGC Standards, Middlesex, UK). MFE-280 endometrial epithelial adenocarcinoma cells were derived from a poorly differentiated endometrial carcinoma from a 78-year-old, catalogue no ECACC-98050131 (Public Health England, Salisbury, UK) p68, Lot no 11J030. The human MDA-MB-231 breast adenocarcinoma cell line was originally isolated from pleural effusions of a Caucasian 51-year-old breast cancer patient (ECACC catalogue no. 92020424). The source and authentication of cell lines are described in Supplementary Table 1 (see section on supplementary materials given at the end of this article) using the ICLAC cell line checklist as a template.

Cells were maintained at 37°C, 5% CO_2_ in DMEM supplemented with 1% non-essential amino acids, 2 mM l-glutamine, 10^5^ U/L penicillin, 100 mg/L streptomycin, 1.25 g/L fungizone and 10% heat-inactivated foetal bovine serum (FBS). For experiments, cells were grown for 48 h in phenol red free DMEM supplemented with 10% charcoal stripped FBS (CSFBS). Previous studies in our laboratory had established that the MDA-MB-231 cells did not contain either mRNA or protein encoded by *ESR1*, whereas the Ishikawa cells used in this study contained both ERα mRNA and protein (Collins *et al*. 2009). Comparison of Ishikawa RL95-2 and MFE endometrial cancer cells revealed that endogenous expression of ERα could only be detected in the Ishikawa cells where it was approximately 1:1 with ERβ5 (Supplementary Fig. 2): failure to detect ERα in the other cells would be consistent with loss of expression in less differentiated cancer cells (Collins *et al*. 2009).

### Transient transfections to establish cell lines expressing different receptor ratios

Adenoviral constructs expressing full-length ERα, ERβ1 and ERβ5 cDNAs were prepared as described previously (Bombail *et al.* 2010). In order to generate proteins with fluorescent protein tags for FRAP analysis (see below) full-length cDNAs encoding human ERα and ERβ5 were subcloned between the EcoRI and BamHI restriction sites in plasmid vectors expressing yellow fluorescent protein (pEYFP-C1) or cyan fluorescent protein expression vector (pECFP-C1) (Clontech, Mountain View, CA, USA). Inserts (YFP/CFP-receptor) were subcloned into the pDC315 shuttle vector (Microbix) recombined into the adenoviral genome (pBHGLOx deltaE1, Cre, Microbix) and used to generate high titre stocks as previously described (Bombail *et al*. 2010). To generate an Ad-ERE-Luc reporter the cDNA from a plasmid construct containing a 3xERE-tk-luciferase reporter gene that was a kind gift from Professor DP McDonnell (Hall & McDonnell 1999) (Duke University NC, USA) was sub-cloned into an adenoviral vector and particles purified as described earlier (Bombail *et al*. 2010).

To establish cells with expression of ERα, ERβ1 and ERβ5 MDA-MD-231 and Ishikawa cells were plated at 1 × 10^5^ cells/mL in phenol red free DMEM with 10% CSFCS for 24 h prior to infection with adenovirus expressing each receptor at multiplicity of infection (MOI) of 50 for 4 h before replacing the media with serum free DMEM. The cells were cultured for 24 h for RNA expression and 48 h for protein expression. To establish Ishikawa cells with an ERβ5 > ERα ratio adenovirus expressing ERβ5 was used at a MOI of 75 and ERα was knocked down using a Silencer Select Predesigned siRNA (Ambion/Life). Cells were seeded at 1 × 10^5^ cells/mL and grown to 60–70% confluence before being transfected with Lipofectamine RNAiMAX (Life) and 15 pmol of siRNA per well. Cells were incubated for 48 h for mRNA expression and 72 h for protein expression. Cells were stimulated with vehicle control (ethanol), E2 10^−8^ M (Sigma) or 10^−8^ M of the ERα-selective agonist PPT (4,4′,4″-(4-propyl-[1*H*]-pyrazole-1,3,5-triyl)*tris*phenol, Tocris; Meyers *et al.* 2001) for 8 h.

### RNA extraction and TaqMan quantitative RT-PCR

RNA extraction from tissues or cells was performed as described in Collins *et al*. (2009): RNA concentration and purity was measured using the NanoDrop (LabTech International, Lewes, UK) and standardised to 100 ng/µL for all samples. RT was performed using 100 ng of RNA with 0.125× Superscript Enzyme in 1× VILO reaction mix (Life, Paisley, UK) at 25°C for 10 min, followed by 42°C for 60 min and finally 85°C for 5 min. Quantitative PCR was performed using probes for genes of interest from the Universal Probe Library (Roche Diagnostics) and specific primers as detailed in Collins *et al*. (2009).

### Double fluorescent immunohistochemistry on tissue sections

Tissue sections were subjected to antigen retrieval in citrate buffer pH6 and processed according to standard laboratory protocols. Sections were first incubated with mouse monoclonal ERβ5 (clone 5/25. BioRad, cat no. MCA4676T) diluted 1:200 in normal goat serum (NGS) overnight at 4°C, followed by goat anti-mouse peroxidase fab (Abcam) 1:500 in serum for 30 min at room temperature and finally incubated with Tyramide Fluorescein (PerkinElmer) at 1:50 in kit diluent for 10 min. Antibody elution was carried out by boiling sections in citrate buffer for 2.5 min followed by 30 min rest, incubated in NGS for 30 min at RT, blocked by streptavidin/biotin following manufacturer’s instructions (Vector, Peterborough, UK). Sections were washed and incubated with ERα mouse monoclonal (Vector, cat no. VP-E614) at 1:80 in NGS overnight at 4°C. Slides were incubated with goat anti-mouse biotinylated (Abcam) at 1:500 in serum for 30 min at RT, followed by Streptavidin Alexa fluor 546 (Molecular Probes) 1:200 in PBS for 1 h. Sections were washed, counterstained with DAPI (Sigma) at 1:1000 in PBS for 10 min before finally mounting in Permafluor (PerkinElmer). All washes between antibodies were carried out three times in TBS. Full details of antibodies used in the study are provided in Supplementary Table 2.

### Luciferase reporter assays

The first set of experiments consisted of Ishikawa and MDA-MD-231 cells (either uninfected) or infected with adenovirus containing constructs for ERα or ERβ5 alone, or both ERα and ERβ5 at MOI of 50. In a second set of experiments Ishikawa cells were stably infected with ERβ5 at MOI of 75 (to overexpress ERβ5) or transfected with a siRNA specific for ERα (using reagents in siERα assay ID s4824 silencer select, Invitrogen) allowing the functional impact of different ratios of ERα to ERβ5 to be examined. In both experiments cells were plated at 1 × 10^5^ cells/mL in 24-well tissue culture plates in DMEM with 10% CSFBS and cultured for 24 h before infection with Ad-ERE-Luc vector at MOI of 50; media was replenished after 4 h. Cells were incubated for 24 h prior to treatment with vehicle control (ethanol), E2 10^−8^ M (Sigma) or PPT 10^−8^ M (Tocris). Luciferase activities were determined using Bright-Glo luciferase reagents according to the manufacturer’s instructions (Promega).

### Fluorescence recovery after photobleaching (FRAP)

Cells cultured on 35 mm cover slips in 60 mm plates (Mat-Tek) at 1 × 10^5^ cells/mL were infected with each of the viral constructs (MOI 50) for 24 h prior to live cell imaging. Cells were maintained in 2.5% HEPES/PBS solution on a heated stage at 37°C. Only cells with relatively low levels of fluorescence were used in the FRAP experiment to avoid problems associated with overexpression and the bulk averaging of large numbers of nuclei.

FRAP was conducted using a Zeiss LSM 510 laser scanning confocal microscope. Images were captured in a 256 X by 100 Y frame through 63× objective lens before and after ligand treatment at 3 s intervals for up to 30 s after bleaching. Bleaching was carried out on a single z-section of the chosen cell (ROI I) with excitation of the Argon 12 laser (488 and 514 nm) and emission via the 530–600 band pass yellow filter. The pinhole was kept open to the maximum and the number of iterations kept at 100. The fluorescence intensity data were normalised for each cell and used for in a non-linear regression model, *Y* = *Y*_max_ × (1 − *e* ^−^ *^Kx^*) (GraphPad Prism 4), where the regression coefficient *r* ^2^ was typically 0.95. The *Y*_max_ and half-life of recovery values (0.69/*K*) were averaged for at least 20 cells per treatment.

### Statistical analysis of FRAP measurements

The bleached area was designated Region of Interest I (ROI I). A second unbleached region in the same cell (ROI II) was used to normalise the bleached area. A third region (ROI III) was chosen outside the nucleus of interest to ensure the bleaching effect was focused on ROI I only. Fluorescence intensity of the bleached region over the time course of scans were normalised against those of ROI II to account for the differences in immunofluorescent levels throughout the cell nucleus. All scanned images post bleach were normalised against the pre-bleached state to derive the percentage recovery (and to allow for differences in actual strength of bleaching between cells). The first image post bleach was subsequently normalised to 0 and recovery rates defined against this value. Variability between cells was resolved by normalising time at bleaching to 0 and successive scan times measured against this. A non-linear regression curve fit was carried out on the resultant figures. This generated the values of *Y*_max_ (maximum level of recovery at which values reach a plateau) and half-time (time taken in seconds to reach half of the *Y*_max_). Unpaired *t*-tests of the regression statistics were carried out to compare these between the treated versus ligand-stimulated cells. Significant differences were noted as those with *P* ≤ 0.05.

## Results

### ERβ5 mRNA and protein are expressed in both normal endometrium and endometrial adenocarcinomas

**Figure 1:**
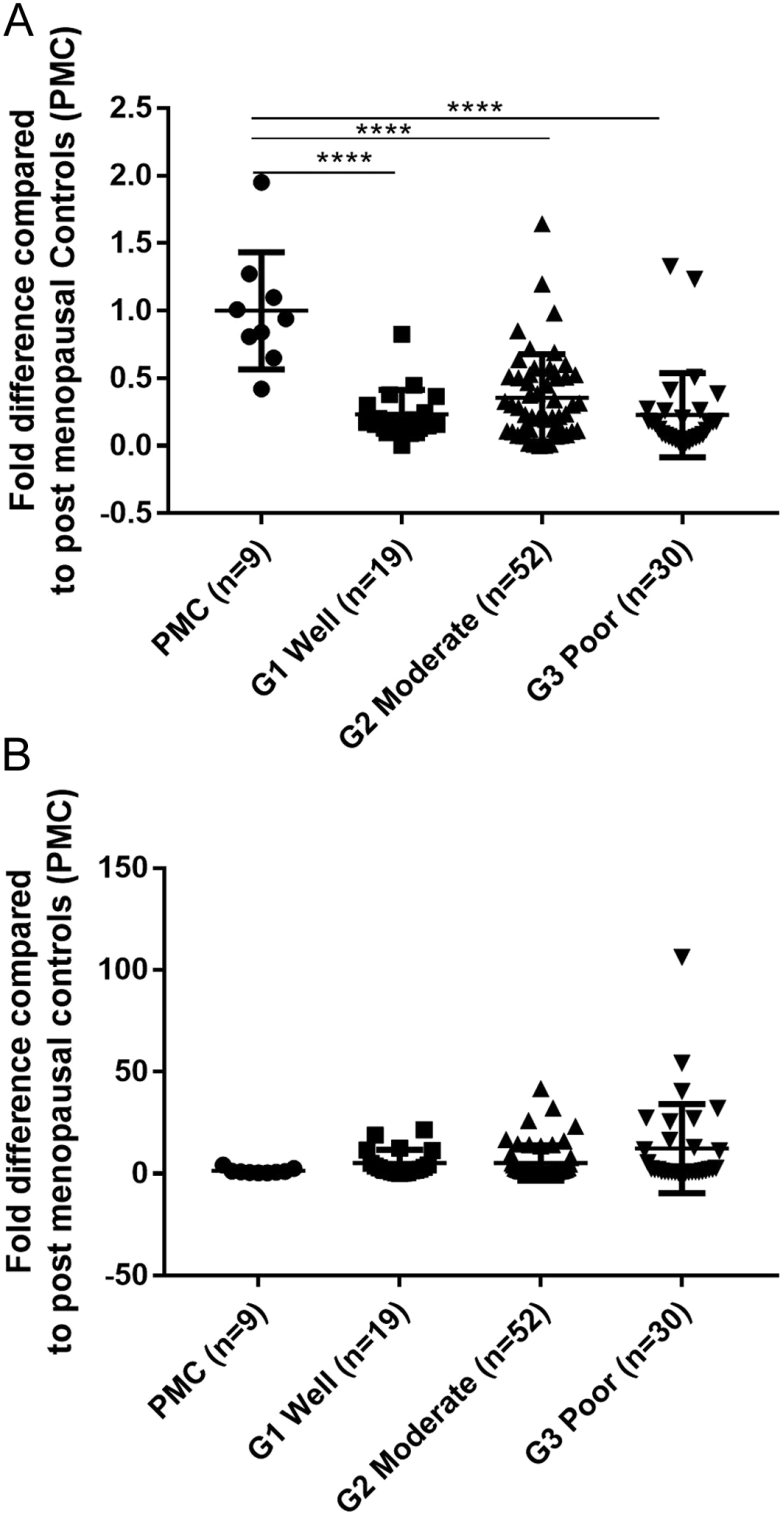
Detection of mRNAs for ERα and ERβ5 in endometrial cancers. Expression of ERα/ERβ5 mRNA is altered in women with endometrial cancer compared to post menopausal controls (PMCs). Expression of ERα mRNA (A) and ERβ5 mRNA (B) in PMCs (*n* = 9), G1 well differentiated (*n* = 19), G2 moderately differentiated (*n* = 52) and G3 poorly differentiated (*n* = 30). Total RNA for ERα in G1, G2, and G3 (*P* < 0.0001) were significantly lower than PMCs. Expression of ERβ5 mRNA appeared to increase in G3 compared to PMC but did not reach significance. Results are expressed as fold difference compared to PMCs with statistical analysis performed by one-way ANOVA with Tukey’s *post hoc* test, *P < 0.05, **P < 0.01, ***P < 0.001, ****P < 0.0001.

Messenger RNAs for both ERα and ERβ5 were detected in endometrial samples from post-menopausal women (PMC, Fig. 1A and B). Expression levels of ERα mRNA were significantly lower in cancers graded as G1 well defined (*P* < 0.01), G2 moderately defined (*P* < 0.01) or G3 poorly defined (*P* < 0.001) than in PMC (Fig. 1A). ERβ5 mRNA expression appeared to be higher in the cancers than the PMC tissue although the wide variation between patients meant this did not reach statistical significance (Fig. 1B). These findings extend those previously reported on a subset of 30 of these 101 endometrial cancer samples (Collins *et al*. 2009).

### Immunoflourescent co-staining of ERβ5 and ERα identified epithelial cells which express both proteins in type I endometrial cancers

**Figure 2:**
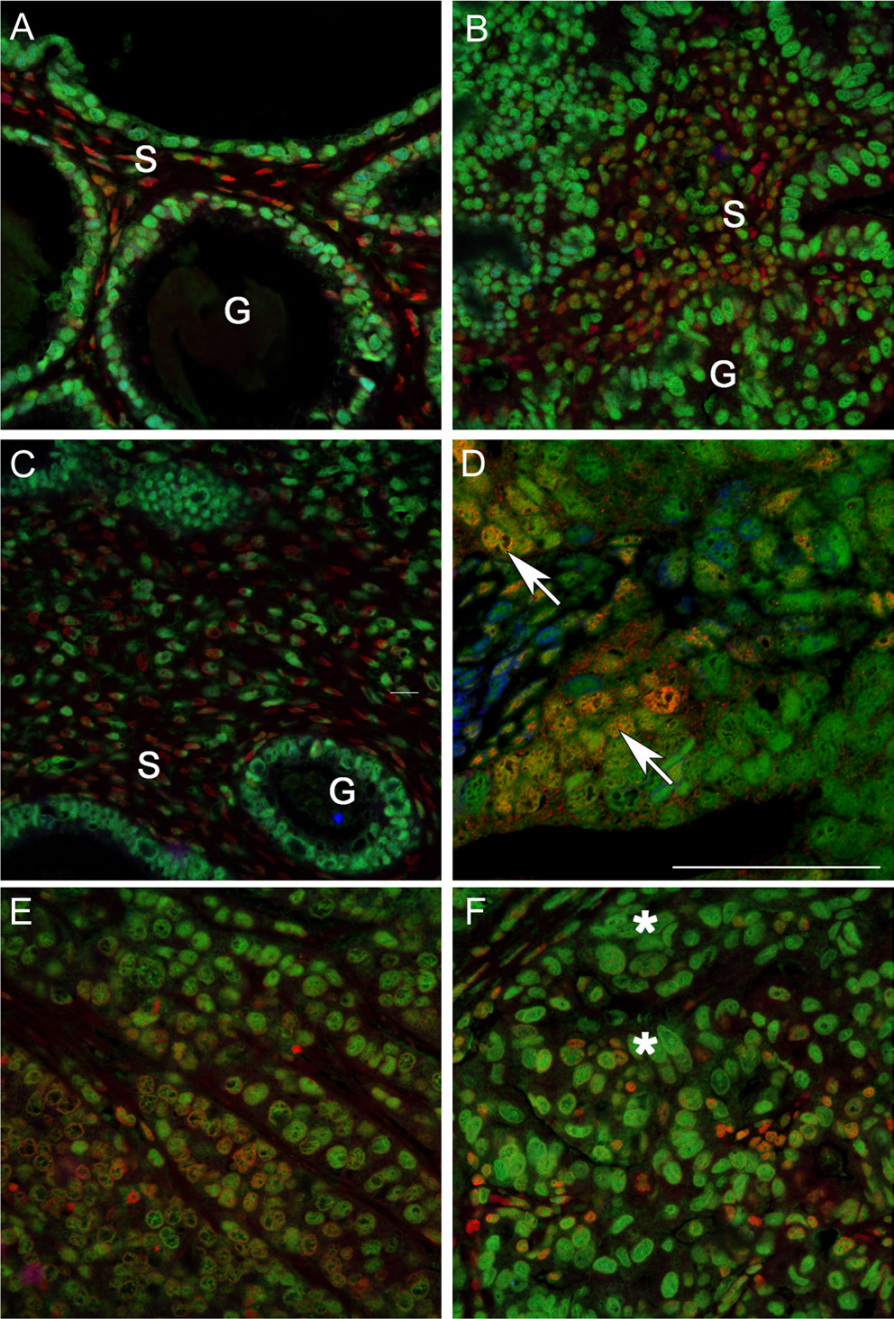
Co-localisation of ERα and ERβ5 in stage 1 endometrial adenocarcinomas identifies variable co-expression of both proteins in a subset of epithelial cells. Examples of staining in endometrial cancer tissues classified by a pathologist as G1 well (A and B), G2 moderately (C and D) or G3 poorly (E and F) differentiated. Note glands (G) surrounded by a single layer of epithelial cells could be identified in well and some moderately differentiated tissue associated with a stromal compartment (S) containing fibroblasts (s). The architecture of the poorly differentiated cancers was less organised and dominated by epithelial cells. Intense immunostaining for ERβ5 (green, asterisks) as well as evidence of co-expression of ERα (yellow-red, arrows) was detected in epithelial cells.

**Figure 3:**
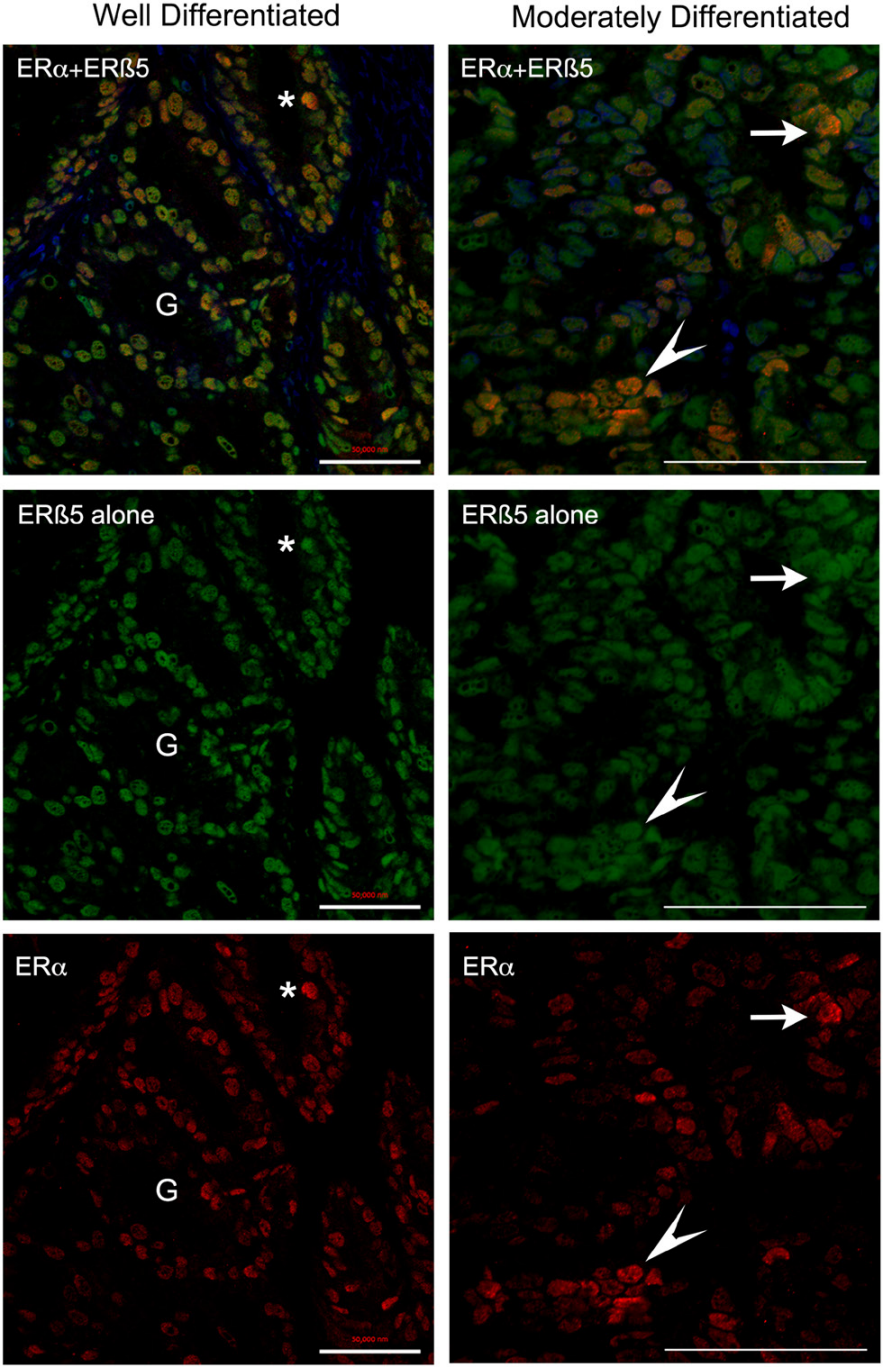
Confocal imaging identifed epithelial cells in endometrial cancers with variable amounts of ERα and ERβ5 proteins. Confocal images typical of endometrial cancers classified as well or moderately differentiated are illustrated showing merged (top panel) and individual channels for ERβ5 (green, middle) and ERα (lower red). The intensity of immunostaining for ERβ5 appeared similar between different nuclei within each of these samples whereas the amount of protein in nuclei stained with an antibody specific for ERα (red) revealed a range of intensities from low to high with the latter identifed by yellow/orange staining in the merged image (examples * and arrowhead). Scale bars 50 µm.

Fluorescent co-staining with antibodies specific for ERα or ERβ5 identified cells expressing one (green, red) or both (yellow/orange) proteins in stage I endometrial cancers (Fig. 2). In samples of well- and moderately differentiated cancers there was a well-defined epithelial layer surrounding gland-like structures (G) which had intense immunostaining for ERβ5 (green nuclei, Fig. 2A, B and C), but within the stroma there were cells that appeared to express ERα (red) alone (fibroblast-like shape) (Fig. 2A, B and C). In samples with a more disorganised tissue architecture (Fig. 2D, E and F) there was no distinct gland structure but coexpression of ERβ5 and ERα was readily detected (yellow/orange cell nuclei). When the green (ERβ5) and red (ERα) channels were separated it was apparent that the intensity of immunostaining for ERα in epithelial cells was variable, whereas ERβ5 appeared more uniform resulting in variable ratios of ERα:ERβ5 in individual epithelial nuclei (Fig. 3).

In endometrium from postmenopausal women both ERα and ERβ5 proteins were detected with evidence of co-expression in some epithelial cells lining the glands, whereas those lining the lumen appeared to lack ERα (Supplementary Fig. 1).

### ERβ5 enhances E2-dependent activation of an ERE reporter gene

**Figure 4:**
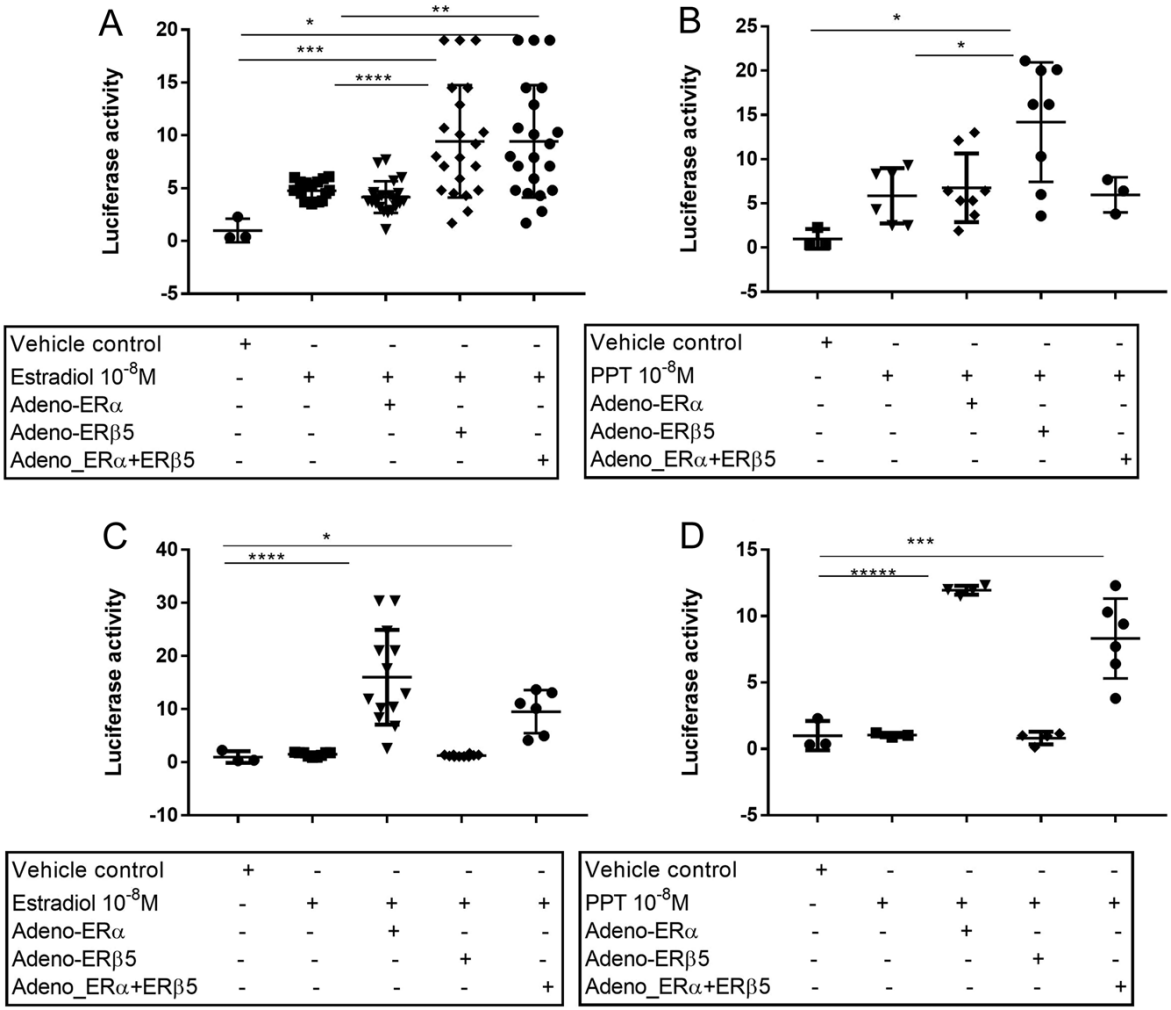
Impact of ERβ5 on expression of an ERE-luciferase reporter gene in Ishikawa and MDA-MD-231 cells. Overexpression of ERβ5 significantly increased the ERE-luciferase activity in response to E2 (***P < 0.001) and PPT (*P < 0.05) in Ishikawa cells (A and B). Increased expression of the reporter response to E2 (C) or PPT (D) was detected in MDA-MD-231 cells transfected with ERα (****P < 0.0001) but not with ERβ5 alone. The number of replicates ranged from a minimum of four on triplicate wells and statistical analysis was performed by one-way ANOVA with Tukey’s *post hoc* test, *P < 0.05, **P < 0.01, ***P < 0.001, ****P < 0.0001.

To investigate if ERβ5 expression altered oestrogen responsiveness, two cell lines were used: endometrial Ishikawa cells that contained both ERβ5 and ERα mRNAs (ratio ~1:1) and MDA-MB-231 breast cancer cells which were ERα negative and had only very low levels of endogenous ERβ5 mRNA (Supplementary Fig. 3). Like MDA-MB-231 two endometrial cancer cell lines (RL92-2, MFE) that were evaluated also lacked endogenous ERα mRNAs but had much higher concentrations of ERβ5 which made them unsuitable for the transfection study. In addition to these wild-type cell lines transfections of each cell line were undertaken using adenoviral vectors containing ERα (Ad-ERα) or ERβ5 (Ad-ERβ5) alone or in combination. In response to treatment with E2, or the ERα-selective agonist PPT (Meyers *et al*. 2001), WT Ishikawa cells significantly increased expression of a luciferase reporter gene under the control of an ERE response element compared to vehicle (Fig. 4A). Tranfection with Ad-ERβ5 significantly increased luciferase expression in response to E2 (Fig. 4A) or PPT (Fig. 4B) compared with WT cells or those transfected with Ad-ERα (Fig. 4B). Co-transfection of cells with Ad-ERα + AdERβ5 did not increase expression of luciferase in the Ishikawa cells beyond that of the cells infected with ERβ5 alone in response to E2 (Fig. 4A) and appeared to blunt the response to PPT (Fig. 4B). In line with expectations, MDA-MD-231 cells did not upregulate expression of the ERE-luc reporter in response to E2 or PPT unless they were infected with Ad-ERα either alone or in combination with Ad-ERβ5 (Fig. 4C and D). In contrast to Ishikawa cells transfection with Ad-ERβ5 had no impact on expression of the ERE-luc reporter consistent with MDA-MD-231 cells lacking endogenous ERα (Fig. 4C and D).

**Figure 5:**
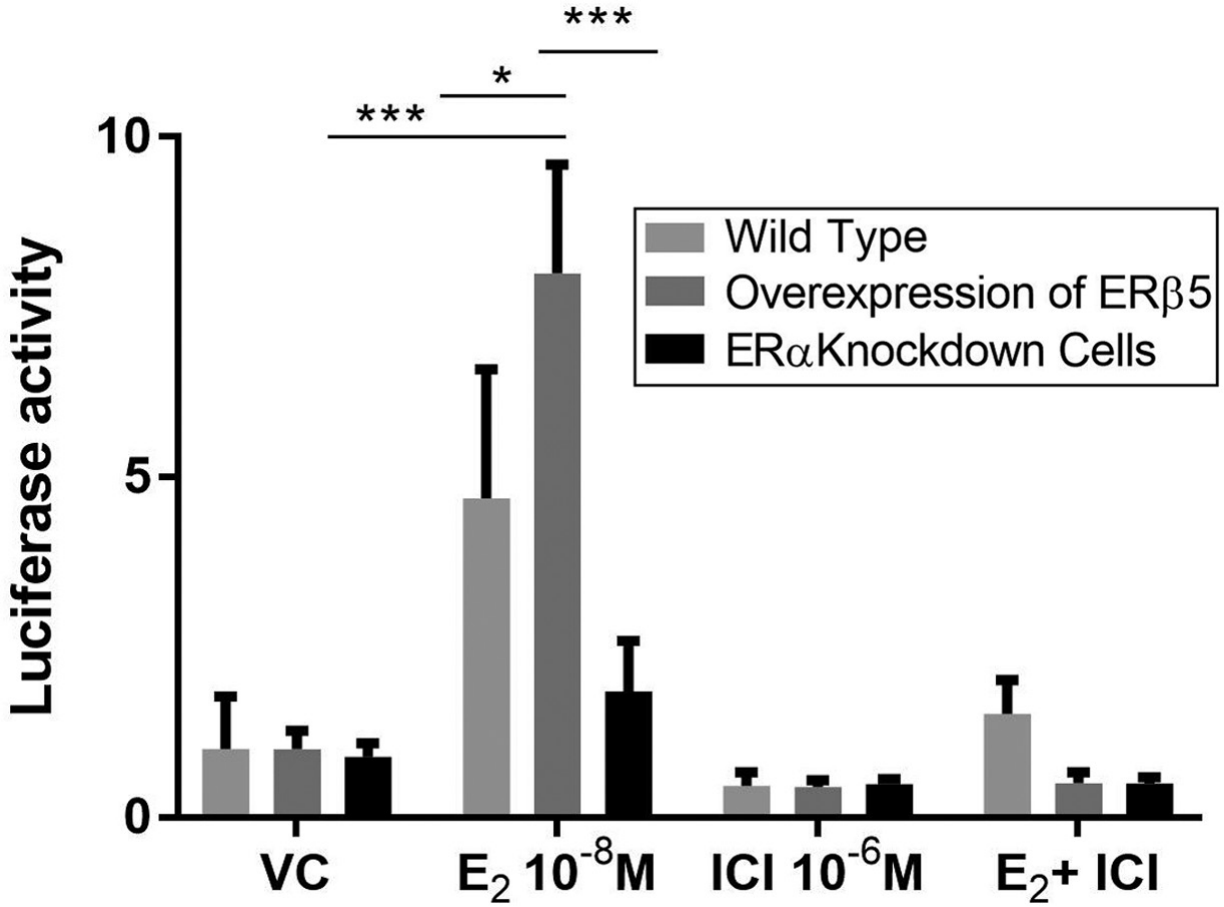
ERα plays a critical role in E2-dependent reporter gene activity in Ishikawa cells expressing ERβ5. Overexpression of ERβ5 in ERα^pos^ Ishikawa cells (ratio mRNAs ERβ5: ERα = 1.5:1) resulted in a significant increase in reporter gene compared to cells treated with vehicle (24 h ± E2). Targeted knockdown of ERα abrogated response to E2. Results are displayed as fold difference compared to vehicle: triplicate experiments performed in triplicate wells. Statistical analysis was performed by one-way ANOVA with Tukey’s *post hoc* test, *P < 0.05, **P < 0.01, ***P < 0.001, ****P < 0.0001.

To extend these studies ERE reporter activation in Ishikawa cells that expressed three different ratios of mRNAs encoded by the receptors were compared: (a) WT cells ~1:1 ratio (ERα:ERβ5), (b) cells infected with Ad-ERβ5 (ratio ERβ5:ERα ~1.5:1), (c) cells depleted of ERα using siRNA-mediated knockdown (ERβ5:ERα ~2.5:1). Protein knockdown resulting in reduced expression of ERα were confirmed by Western blot (Supplementary Fig. 4). Consistent with earlier findings WT cells and those with enhanced expression of ERβ5 both increased expression of the ERE-luc reporter in response to E2 with a significant increase in the Ad-ERβ5 cells compared to WT (Fig. 5). The importance of ERα was confirmed by siRNA knockdown and by incubation of the cells with the anti-oestrogen ICI (Fig. 5).

### FRAP analysis of YFP-ERβ5 reveals altered mobility in response to E2 in Ishikawa cells

As ERE reporter studies suggested that ERβ5 could alter transcriptional activity in Ishikawa cells when co-expressed with ERα, further experiments were performed to explore whether this was associated with formation of ERα/ERβ5 heterodimers.

**Figure 6:**
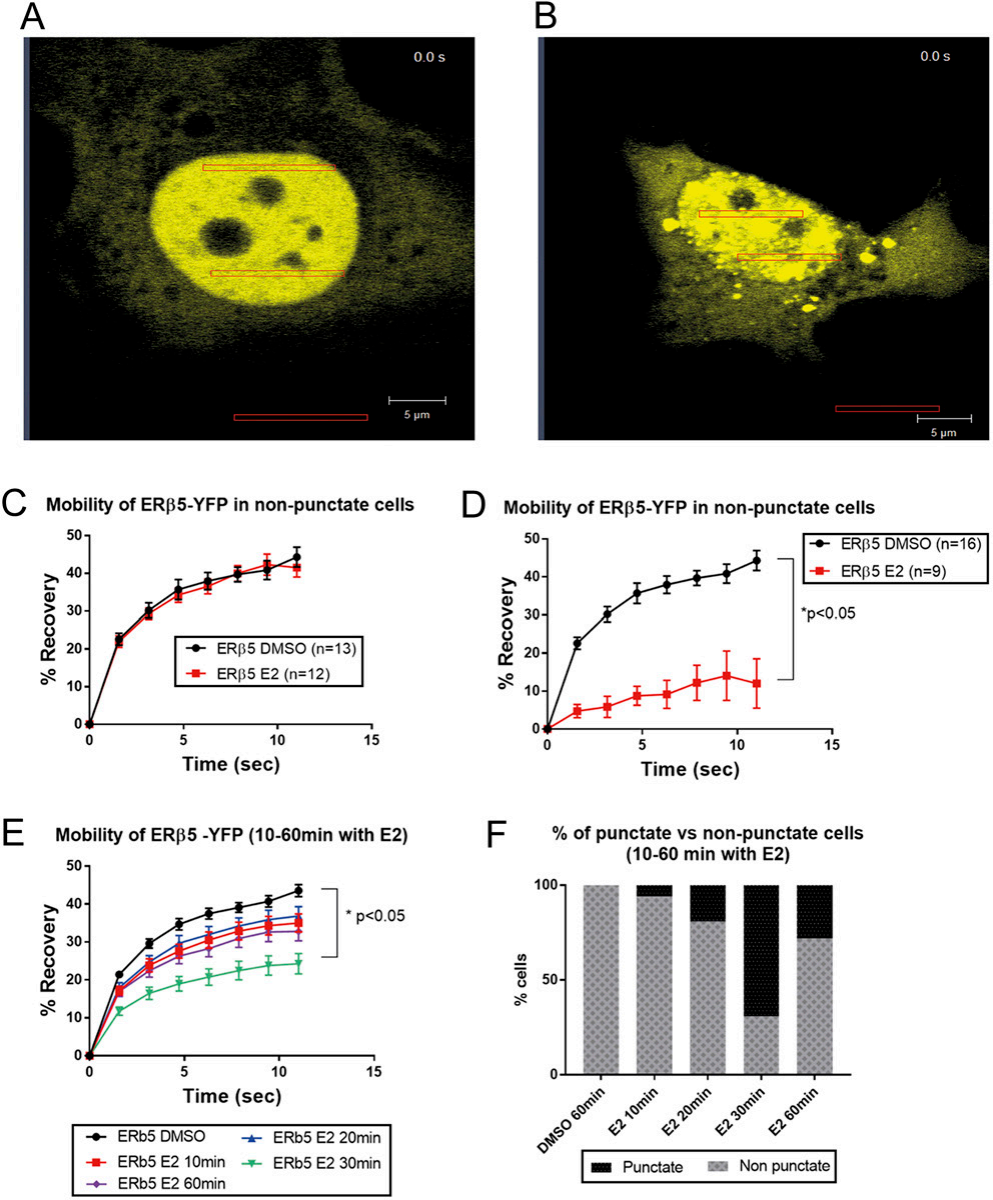
FRAP analysis of YFP-tagged ERβ5 in Ishikawa identifies a population of cells with altered nuclear mobility of ERβ5 in response to E2. Following incubation of ERα^pos^, YFP-ERβ5 Ishikawa cells with E2 two populations of cells were identified: (A) cells in which YFP-tagged ERβ5 was uniformly distributed within the nucleus apart from the nucleolus (dark circles) and (B) cells in which YFP protein was not uniform but appeared to be concentrated in selected regions (hereafter referred to as ‘punctate’). Using the software of the confocal it was possible to determine the mobility of YFP protein within a bleached region of interest (ROI): protein in A cells remained highly mobile regardless of the presence of ligand (C) whereas in B cells addition of E2 resulted in a rapid reduction in mobility (D). Further analysis of the population of cells exhibiting altered mobility (E and F) revealed that the change in mobility following addition of E2 was time-dependent with the highest percentage of punctate cells at 30 min (F). A minimum of 9 to a maximum of 16 individual cells were examined.

Live cell imaging and FRAP were used to explore the dynamics of YFP-tagged ERβ5 in the nuclei of ERα^pos^ Ishikawa and ERα^neg^ MDA-MD-231 cells using established methods (Bombail *et al*. 2010). Following transfection of Ishikawa cells with the majoirty of YFP-ERβ5 protein being detected in the nuclear compartment in line with expectations FRAP analysis revealed that in cells treated with DMSO (vehicle control), this protein was highly mobile (Fig. 6C and D). Addition of E2 resulted in changes in the appearance of some but not all cell nuclei. In one population of cells where there was no evidence of altered mobility in response to E2 (Fig. 6A and C) but in second population of cells incubation with E2 induced a rapid reduction in intra-nuclear receptor mobility and adoption of a ‘punctate’ distribution (Fig. 6B and D). Further detailed analysis of the latter revealed that the punctate appearance was both rapid and transient, peaking ~20 min after introduction of E2 (Fig. 6E and F). Mobility of YFP-ERβ5 in MDA-MD-231 cells was not altered by treatment with E2 even when cells were co-transfected with ERα (Supplementary Fig. 5): these results are consistent with the results obtained using the ERE-luciferase reporter.

## Discussion

Life-time exposure to oestrogens, treatment with drugs with oestrogenic activity, exposure to endocrine disruptors, or oestrogen exposure unopposed by progesterone (for example during the peri-menopause) have all been implicated in rising rates of endometrial cancer (reviewed in Rizner *et al*. 2017, Sanderson *et al*. 2017).

In contrast to the limited data on ERβ5 a large number of publications have suggested that ERβ1, the full-length *ESR2* isoform, which has an intact ligand-binding pocket, acts as a negative modulator of ERα in breast and other cancer cells (Chang *et al.* 2006, ** Zhao *et al*. 2007). A systematic review of evidence from immunohistochemical studies of breast cancers concluded that the positive association between ERβ1 expression and 5-year overall survival was only evident in ERα-positive patients (Liu *et al.* 2016). Structural analyses also suggest ERαβ heterodimers are more stable than ERββ homodimers and conservation of peptides implicated in the heterodimeric interaction in ERβ5 are consistent with historical gel shift studies reporting this variant can dimerise (Poola *et al*. 2005, Chakraborty *et al.* 2012). In a study using single-chain ERs to explore the relative contributions of ERα and ERβ1 to heterodimer activities, Li *et al.* (2004) reported ERα is the functionally dominant partner in ERα/ERβ1 heterodimers. The results of the current study appear to be in agreement with this observation with binding of ligand to ERα essential to the activation of reporter constructs.

Our studies in endometrial cancer tissue are in agreement with other results reporting expression of ERβ5 protein is upregulated in a number of hormone-responsive cancers compared with equivalent non-malignant tissues (Li *et al*. 2015). Smith *et al.* identified different exons (E0K, E0N) in the 5′UTR sequences of *ESR2* transcripts (Smith *et al.* 2010) and showed that the translational efficiency of a GFP reporter gene was higher when the promoter contained the E0N exon sequence. They highlighted the importance of translational regulation in determining expression levels of *ESR2* variants, including ERβ5, in breast cancer cell lines (Smith *et al*. 2010). They also speculated that overexpression of eIF4E could explain an increase in the translational efficiency *ESR2* variants such as ERβ5 in cancer. Although it would be interesting to determine which 5′UTR drives the expression of ERβ5 variant mRNAs in endometrium and whether this is altered in endometrial cancers, this was outside the scope of the current investigation.

In this study we have, for the first time, demonstrated that ERα and ERβ5 proteins are co-expressed in endometrial adenocarcinomas with evidence that most epithelial cells in stage I cancers were immunopositive for ERβ5 but with variable expression of ERα. These results are in agreement with previous findings obtained using a subset of the current samples and single colour staining (Fig. 2 in Collins *et al*. 2009). A paper by Haring *et al.* (2012) has reported that the ratio of ERβ5:ERα mRNA rises in parallel with grade.

As ERβ5 protein is clearly expressed in some endometrial cancers in a pattern that overlaps with that of ERα we used a variety of cell-based methods to explore whether this might alter the response of cells to E2. Studies were conducted in Ishikawa cells which expressed endogenous ERα as well as MDA-MD-231 cells which had no native ERα: significant differences in the impact of overexpression of ERβ5 in these cell backgrounds were apparent when their oestrogen responsiveness was assessed using a reporter gene under the control of an ERE promoter. In the Ishikawa cells overexpression of ERβ5 resulted in a significant *increase* in reporter gene activity in response to either E2 or PPT, an ERα-selective agonist. Further studies using siRNAs confirmed that activation of the reporter gene was ERα dependent. In contrast in MDA-MD-231 cells there was no induction of the ERE reporter in WT cells or those transfected with Ad-ERβ5. A key question arising from these studies was how does ERβ5 increase ERα-dependent ERE activation even though the protein is unable to bind E2? One possible explanation is that it stabilises a conformation of ERα that favours co-activator recruitment. In this study we showed that the ratio between the different receptors makes a difference to activation of the ERE reporter in Ishikawa cells with a ratio of ERβ5:ERα mRNAs of between 1:1 and 1.5:1 able to enhance reporter responses. In MDA-MD-231 cells co-expression of ERβ5 with ERα did not enhance response to E2 or PPT above that of ERα alone. It has been reported that ERβ5 can inhibit ERα-dependent activation of an ERE reporter gene in COS7 cells (Peng *et al*. 2003). Older papers have also reported that greater ratios of ERβ5 (10:1 ERα) resulted in reduced expression of ERα (Poola *et al*. 2005). These contrasting results suggest cell context (availability of cofactors?) as well as the ratios of ER subtypes can alter oestrogen responsiveness but still need to be repeated in a wider range of cell types to validate this hypothesis.

Reporter gene activation is a useful and widely employed read-out of oestrogen response but FRAP is a more powerful tool as it allows for monitoring the mobility of receptor proteins in real time in individual cells. The Mancini group have published a number of elegant studies documenting intranuclear dynamics of fluorescent-tagged ERα protein (Stenoien *et al.* 2000, 2001*a*,*b*). They showed that in the absence of steroid ligand ERα is highly mobile within the nuclear environment and that addition of E2 results in reduced mobility which they suggest reflects enhanced interactions with immobile nuclear proteins (Stenoien *et al.* 2001*b*). In the current study we report novel evidence that the intra-nuclear mobility of YFP-tagged ERβ5 was altered in response to E2 in Ishikawa cells. The time frame of the immobilisation and recovery of the YFP-ERβ5 mirrored that of tagged ERα constructs used in our own and other studies including the redistribution into a ‘punctate’ pattern. ERβ5 lacks amino acids corresponding to Helix 12 in the wild-type ERβ1 protein. It has been reported that these sequences are required for ligand-dependent immobilisation of ERα (Stenoien *et al*. 2001*b*); hence, the formation of a heterodimer with ligand-activated ERα is the most likely mechanism by which this change in ERβ5 mobility is occurring. Notably, in the current study, not all Ishikawa cells transfected with YFP-ERβ5 showed altered intranuclear mobility in response to E2. Immunostaining of cells from cultures of Ishikawa cells used in this study with anti-ERα antibodies (data not shown) revealed variable expression of ERα leading us to conclude reduced mobility of YFP-ERβ5 in E2-treated cells is restricted to those cells that are ERα^pos^. We also noted parallels between these results and those of a previous study using Ishikawa cells in which we detected changes in intranuclear mobility of an FP-tagged construct of an orphan member of the nuclear receptor superfamily ERRβ which like ERβ5 lacks an intact ligand-binding domain (Bombail *et al*. 2010).

We also performed some experiments using MDA-MD-231 breast cancer cells which lacked endogenous ERα. Notably, whilst a change in nuclear mobility of YFP-ERα was detected in response to E2 co-transfection of YFP-ERβ5 and ERα did not result in altered mobility of the YFP-ERβ5 receptor and we speculate that this cell environment did not favour formation of stable heterodimers (Supplementary Fig. 3). These results highlight the importance of using cells with a phenotype that is close to the disease under consideration.

ERβ5 may also have roles in cancer that are independent of ERα. The sequence of the protein contains an intact N terminal domain containing amino acids might be susceptible to phosphorylation by growth factor-dependent pathways resulting in steroid ligand-independent activation. This has not been tested but may provide a mechanistic explanation as to why expression of ERβ5 is associated with worse outcomes in HER2-positive and triple-negative patients (Wimberly *et al*. 2014) and can have an impact on response to chemotherapeutic agent-induced apoptosis (Lee *et al.* 2013).

Recent efforts to expand our understanding of disease progression have used molecular rather than morphological criteria to define subtypes of endometrial cancers. For example, The Cancer Genome Atlas (TCGA) identified four major endometrial cancer groups (1–4): *POLE* mutations, microsatellite instability, copy-number low/microsatellite stable, and copy-number high/‘serous-like’ (Cancer Genome Atlas Research *et al.* 2013). Notably in this analysis the authors identified three robust clusters termed ‘mitotic’, ‘immunoreactive’ and ‘hormonal’ based on their RNA analysis with the hormonal subgroup being composed of endometrioid grade 1/2 tissues exhibiting upregulation of hormone-responsive genes including ESR1 and PR (Cancer Genome Atlas Research *et al.* 2013). In future studies it would be interesting to see whether upregulated expression of ESR2 (including ERβ5) is also associated with this cluster.

In summary, our results provide novel evidence that expression of ERβ5 may increase oestrogen responsiveness of ERα^pos^ in some endometrial cancer cells by forming ERβ5-ERα heterodimers. A limitation of our study is that only one endometrial cancer cell line was used as other lines tested lacked endogenous ERα hence generalisation of the findings to all endometrial cancers requires investigation in other cells as well as integration with the latest genomic datasets. We suggest that expression of ERβ5 should be considered in risk assessment of women with early grade endometrial cancer as this may inform therapeutic strategies.

## Supplementary Material

Supplementary Table 1

Supplementary Table 2

Supplementary Figure 1. Immunoexpression of ERβ5 and ERα in postmenopausal endometrium. Immunostaining identified glandular epithelial cells in postmenopausal endometrium that co-expressed ERβ5 and ERα in (orange/yellow, asterisks) associated with the glands (G). Notably epithelial cells lining the lumen appeared to be predominantly immunopositive for ERβ5 (green). Cells within the stroma were a mixture of immunonegative (blue), ERβ5 positive and ERβ5/ERα double positive.

Supplementary Figure 2. Expression of ERα and ERβ5 mRNAs in endometrial cancer cell lines. Expression of ERα (A) and ERβ5 (B) mRNAs in Ishikawa, RL95 and MFE cells. N= 3 per sample with triplicate. Note endogenous ERα mRNAs were only detectable in Ishikawa cells.

Supplementary Figure 3. Comparison between Ishikawa and MDA-MB-231cells. Note MDA-MB-231 do not contain quantifiable ERα. N=8-10 per sample with triplicate wells.

Supplementary Figure 4. Protein expression in Ishikawa cells in which ratios of ERα and ERβ5 were manipulated using a pool of siRNAs directed against ERα or lentivirus containing ERβ5. Results were generated by quantification on Western blots using STAT1 as a loading control. N=3 for each condition.

Supplementary Figure 5. FRAP analysis of YFP-tagged ERs in MDA breast cancer cells Individual MDA-MB-231 cells infected with adenovirus expressing full length YPF-tagged ERα (A, positive control); (B) YFP-ERβ5 (C) YFP-ERβ5 plus and untagged ERα; Cells were treated with vehicle alone (DMSO) or vehicle containing E2 10-8M. Note analysis of % Recovery of fluorescence after bleaching the ROI identified a significant decrease in nuclear mobility of YFP only in cells infected with ERα-YFP whereas ERβ5-YFP remained highly mobile even when exogenous ERα was introduced into the cells suggesting the cellular context of these cells did not support/sustain hetero-dimerisation.

## Declaration of interest

The authors declare that there is no conflict of interest that could be perceived as prejudicing the impartiality of the research reported.

## Funding

Studies in the corresponding author’s laboratory were supported by MRC Programme Grants G1100356/1 and MR/N024524/1. C F was supported by a MRC funded PhD studentship paid for from core training funds allocated to the MRC Reproductive Sciences Unit.

## Author contribution statement

P T K S designed the study. F C, N I, A E-Z, C F performed the experiments. Original draft of manuscript: P T K S. Revisions and final draft of manuscript: F C, D A G, P T K S.
