# Supplementary material for: The ERβ5 splice variant increases oestrogen responsiveness of ERαpos Ishikawa cells
Source: Endocr Relat Cancer. 2019 Nov 27;27(2):55–66. doi: 10.1530/ERC-19-0291 (PMC6933808; doi:10.1530/ERC-19-0291)
Supplement: Supplementary Table 1 [file supplementary_table_1.pdf]

Cell Line Checklist for Manuscripts and Grant Applications

This checklist is a resource for scientists who write or review manuscripts and/or for grant applications that use cell lines. Cross-contaminated cell lines could give unreliable results if used for research because they no longer correspond to the reported donor tissue and so may not represent the correct species, tissue type or disease state. Such misidentified or false cell lines produce unreliable research data and we urge reviewers to highlight their use wherever possible.

This checklist will help the author or reviewer to look for obvious cell line quality concerns. The checklist may also be used to communicate any quality concerns to be addressed prior to publication or funding.

Manuscript or Grant Information

Title or Manuscript/Grant ID:	ERb5 increases oestrogen responsiveness of Era positive endometrial cancer cells
Cell Lines used:	Ishikawa: Human endometrial adenocarcinoma,<br>ECACC 99040201<br>MDA-MB-231 Human breast adenocarcinoma<br>ECACC 92020424<br>RL95: Human endometrial epithelial cancer<br>ATCC CRL-1671<br>MFE-280: Human endometrial epithelial adenocarcinoma<br>ECACC 98050131
Cell Lines used with Quality Concerns:	

Cell Line Information

**Reporting Requirement**	**Indicate "Yes" or "No" (No includes Not Known)**<br>**Add further comment if required**
Cell line is known to be cross-contaminated or otherwise misidentified:<br>See the ICLAC website for a register of known misidentified cell lines and Recommendation 1) below.	NO
Authentication testing has been performed:<br>The method and results should be listed.<br>See Recommendation 2) below.	Authentication of Ishikawa and MDA-MB-231was performed by STR analysis (see attached method and results)<br>RL95 and MFE-280authenticated by Eurofins (Ebersberg, Germany) using PCR-single-locus-technology
Human cell lines: STR profile is available with the manuscript/grant application:<br>See Recommendation 2) below.	**DNA Profile: Ishikawa**<br>CSF1PO: 11,12<br>D13S317: 9,12<br>D16S539: 9<br>D5S818: 10,11<br>D7S820: 9,10<br>THO1: 9,10<br>TPOX: 8

<table>
<tr><td></td><td>vWA: 14,17

DNAProfile:MDA-MB-231
CSF1PO: 12,13
D13S317: 13
D16S539: 12
D5S818: 12
D7S820: 8,9
THO1: 7,9.3
TPOX: 8,9
vWA: 15,18</td></tr>
<tr><td>Mycoplasma testing has been performed:
The method and results should be listed.</td><td>Mycoalert ™ Mycoplasma Detection Kit (Lonza, LT07-118).
Absorbance read on CLARIOstar Plus (BMG Labtech)
Positive and negative controls included with each test. Cells routinely tested every 6 months.
A ratio above 1 indicates mycoplasma positive.

Table below is representative of results routinely detected.
<table>
<tr><th>Cell line</th><th>Passage number</th><th>Reading A</th><th>Reading B</th><th>Reading B/Reading A</th><th>Result</th></tr>
<tr><td>MDA-MB-231</td><td>P36</td><td>115</td><td>46</td><td>0.4</td><td>Negative</td></tr>
<tr><td>Ishikawa</td><td>P17</td><td>91</td><td>67</td><td>0.736</td><td>Negative</td></tr>
<tr><td>MDA-MB-231</td><td>P40</td><td>68</td><td>32</td><td>0.471</td><td>Negative</td></tr>
<tr><td>Ishikawa</td><td>P21</td><td>120</td><td>68</td><td>0.567</td><td>Negative</td></tr>
<tr><td>RL95</td><td>P68</td><td>176</td><td>108</td><td>0.614</td><td>Negative</td></tr>
<tr><td>MFE-280</td><td>P72</td><td>138</td><td>120</td><td>0.870</td><td>Negative</td></tr>
<tr><td>Positive control</td><td></td><td>106</td><td>2439</td><td>23.01</td><td>Positive</td></tr>
<tr><td>Negative control</td><td></td><td>163</td><td>17</td><td>0.104</td><td>Negative</td></tr>
</table></td></tr>
<tr><td>Source for cell line is listed:
The catalogue number should be included if obtained from a cell line repository.
See Recommendation 3) below.</td><td>Ishikawa: Human endometrial adenocarcinoma, ECACC 99040201
MDA-MB-231 Human breast adenocarcinoma
ECACC 92020424
RL95: Human endometrial epithelial cancer
ATCC CRL-1671
MFE-280: Human endometrial epithelial adenocarcinoma
ECACC 98050131</td></tr>
<tr><td>RRID Number for cell line is listed:
The Resource Identification Initiative (RRID) is meant to help researchers cite the important resources used in</td><td>Ishikawa (RRID:CVCL_2529)
MDA-MB-231 (RRID:CVCL_0062)
MFE-280 (RRID:CVCL_1405)</td></tr>
</table>

scientific papers.<br>See Recommendation 4) below.	RL95-2 (RRID:CVCL_0505)
Sufficient information is given to replicate experiments using the cell line:<br>See Recommendation 5) below.	Ishikawa passage 16<br>MDA-MB-231 passage 35<br>MFE-280 P68<br>RL95-2 P57<br>Cells passaged a maximum of 10 times

Recommendations

1) ICLAC recommends that false cell lines (misidentified cell lines with no known authentic stock) should not be used. ICLAC's register of misidentified cell lines can be found at http://iclac.org/databases/cross-contaminations.

2) ICLAC recommends that authentication testing should always be performed on established cell lines regardless of the application; the test method and results should be included in the Materials and Methods section. Testing should be done, at minimum, at the beginning and end of experimental work.

   For human cell lines, short tandem repeat (STR) profiling should be performed and compared to results from donor tissue, or to online databases of human cell line STR reference profiles.

   More information can be found in the published Standard: ANSI/ATCC ASN-0002-2011 Authentication of Human Cell Lines: Standardization of STR Profiling. ANSI eStandard Store.

   For non-human cell lines, best practice will vary with the species being tested. At minimum, species should be confirmed using an appropriate method such as karyotyping, isoenzyme analysis, or mitochondrial DNA typing (DNA barcoding).

   More information on authentication testing can be found at http://iclac.org/references/.

3) It will be helpful for the reader if authors can include a reference, to provide more information on the cell line's establishment and characterization. However, not all cell lines have this information available in the public domain.

4) Cell line RRIDs are assigned through a collaboration between Cellosaurus and the Resource Identification Initiative. RRIDs can be found by searching for cell lines at https://web.expasy.org/cellosaurus/

5) This information may include the growth medium used, including additives; any additional growth requirements, including special substrates and gas mixtures; and the passage number or population doubling level (PDL) used for experimental work.

   Passage number is important when working with early passage or finite cultures, or cell lines where changes in phenotype have been documented with increasing passage. ICLAC recommends that laboratories freeze down stocks when they first receive a cell line and set a limit (e.g. 20 passages) to avoid overpassaging. More information can be found at http://iclac.org/resources/advice-scientists/

Notes or Further Comments
