# Supplementary material for: The ERβ5 splice variant increases oestrogen responsiveness of ERαpos Ishikawa cells
Source: Endocr Relat Cancer. 2019 Nov 27;27(2):55–66. doi: 10.1530/ERC-19-0291 (PMC6933808; doi:10.1530/ERC-19-0291)
Supplement: Supplementary Table 2 [file supplementary_table_2.pdf]

Information on Antibodies

Antibody Name	Supplier/Cat number	Target	Species raised, monoclonal/polyclonal	Positive controls	Dilution used following titre optimisation	Comparison with mRNA levels
ERα	Vector/VP-E614<br><br>Clone:ER6F11<br><br>Lot number N/A	Recombinant protein of the full length alpha form of the human estrogen receptor molecule.	Mouse monoclonal	Proliferative endometrium	1:80	√
ERβ5	BioRad/MCA4676T<br><br>Clone: 5/25<br><br>Lot number:080110	Tuberculin conjugated synthetic peptide LLSHVRHARYAP derived from the C-terminus of human ERB5	Mouse Monoclonal	Colorectal cancer	1:200	√
