# Supplementary figures and images for: The ERβ5 splice variant increases oestrogen responsiveness of ERαpos Ishikawa cells
Source: Endocr Relat Cancer. 2019 Nov 27;27(2):55–66. doi: 10.1530/ERC-19-0291 (PMC6933808; doi:10.1530/ERC-19-0291)

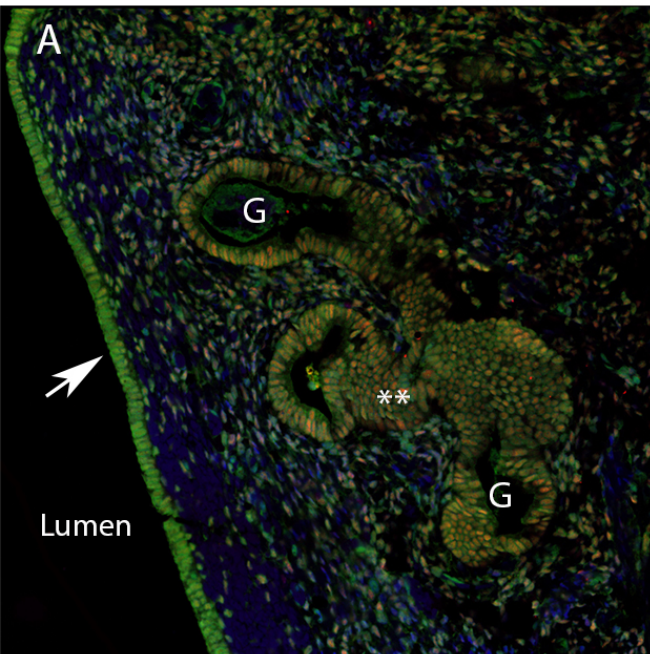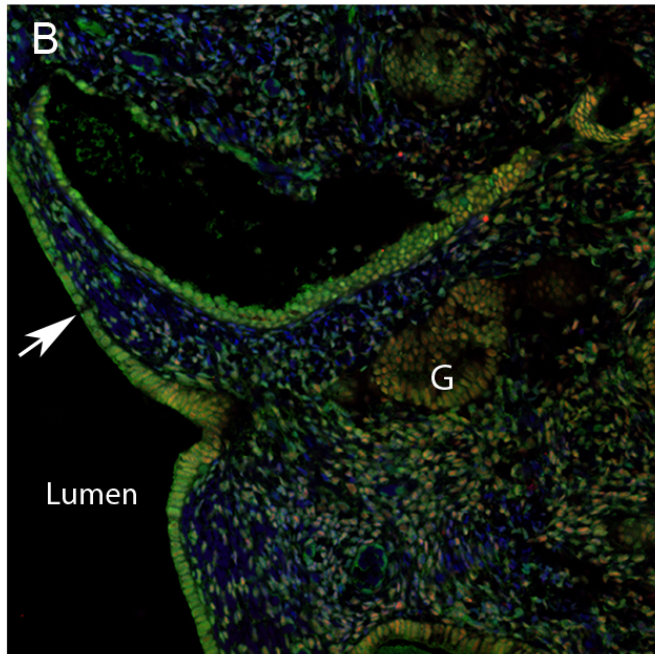

Supplement: Supplementary Figure 1. Immunoexpression of ERβ5 and ERα in postmenopausal endometrium. Immunostaining identified glandular epithelial cells in postmenopausal endometrium that co-expressed ERβ5 and ERα in (orange/yellow, asterisks) associated with the glands (G). Notably epithelial cells lining the  [file supplementary_figure_1.pdf]

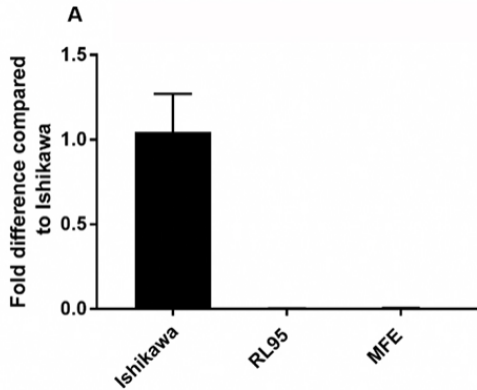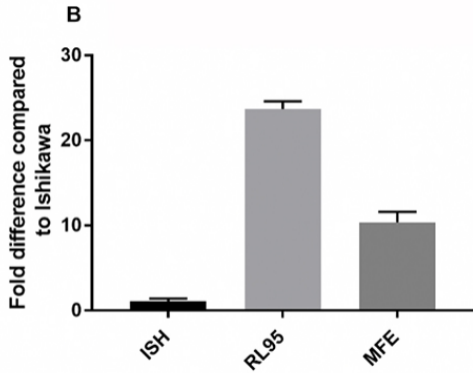

Supplement: Supplementary Figure 2. Expression of ERα and ERβ5 mRNAs in endometrial cancer cell lines. Expression of ERα (A) and ERβ5 (B) mRNAs in Ishikawa, RL95 and MFE cells. N= 3 per sample with triplicate. Note endogenous ERα mRNAs were only detectable in Ishikawa cells. [file supplementary_figure_2.pdf]

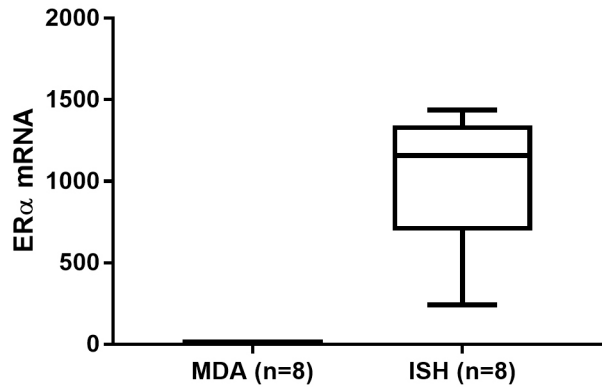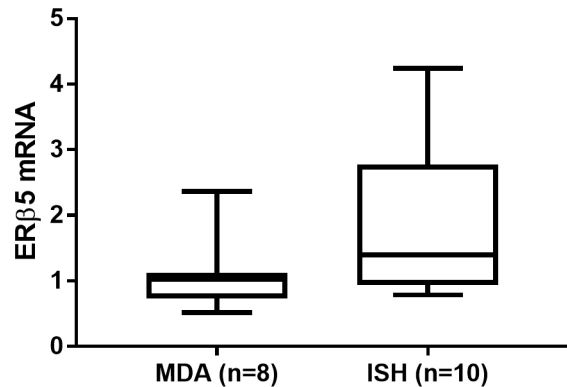

Supplement: Supplementary Figure 3. Comparison between Ishikawa and MDA-MB-231cells. Note MDA-MB-231 do not contain quantifiable ERα. N=8-10 per sample with triplicate wells. [file supplementary_figure_3.pdf]
