# Supplementary material for: The ERβ5 splice variant increases oestrogen responsiveness of ERαpos Ishikawa cells
Source: Endocr Relat Cancer. 2019 Nov 27;27(2):55–66. doi: 10.1530/ERC-19-0291 (PMC6933808; doi:10.1530/ERC-19-0291)
Supplement: Supplementary Figure 4. Protein expression in Ishikawa cells in which ratios of ERα and ERβ5 were manipulated using a pool of siRNAs directed against ERα or lentivirus containing ERβ5. Results were generated by quantification on Western blots using STAT1 as a loading control. N=3 for each condition. [file supplementary_figure_4.pdf]

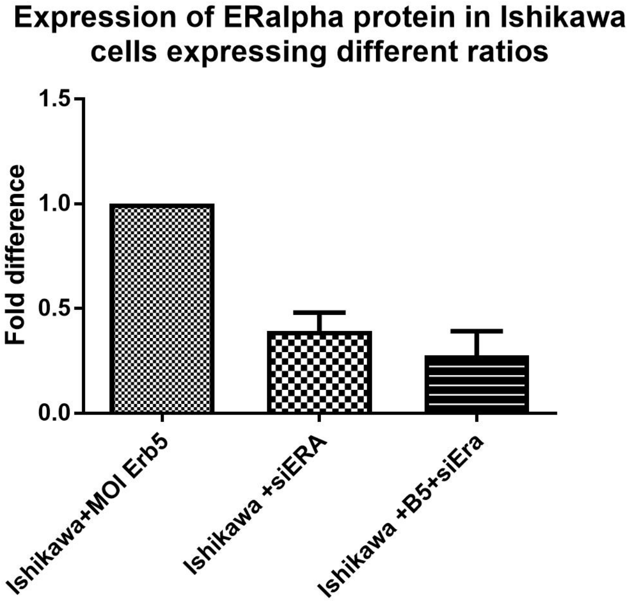
Expression of ERalpha protein in Ishikawa cells expressing different ratios
Fold difference
1.5
1.0
0.5
0.0
Ishikawa+MOI Erb5
Ishikawa +siERA
Ishikawa +B5+siEra

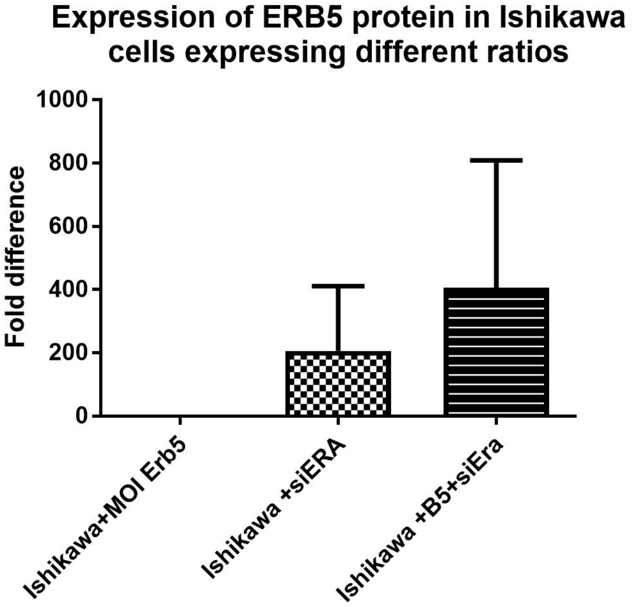
Expression of ERB5 protein in Ishikawa cells expressing different ratios
Fold difference
1000
800
600
400
200
0
Ishikawa+MOI Erb5
Ishikawa +siERA
Ishikawa +B5+siEra
