# Supplementary material for: The ERβ5 splice variant increases oestrogen responsiveness of ERαpos Ishikawa cells
Source: Endocr Relat Cancer. 2019 Nov 27;27(2):55–66. doi: 10.1530/ERC-19-0291 (PMC6933808; doi:10.1530/ERC-19-0291)
Supplement: Supplementary Figure 5. FRAP analysis of YFP-tagged ERs in MDA breast cancer cells Individual MDA-MB-231 cells infected with adenovirus expressing full length YPF-tagged ERα (A, positive control); (B) YFP-ERβ5 (C) YFP-ERβ5 plus and untagged ERα; Cells were treated with vehicle alone (DMSO) or vehicl [file supplementary_figure_5.pdf]

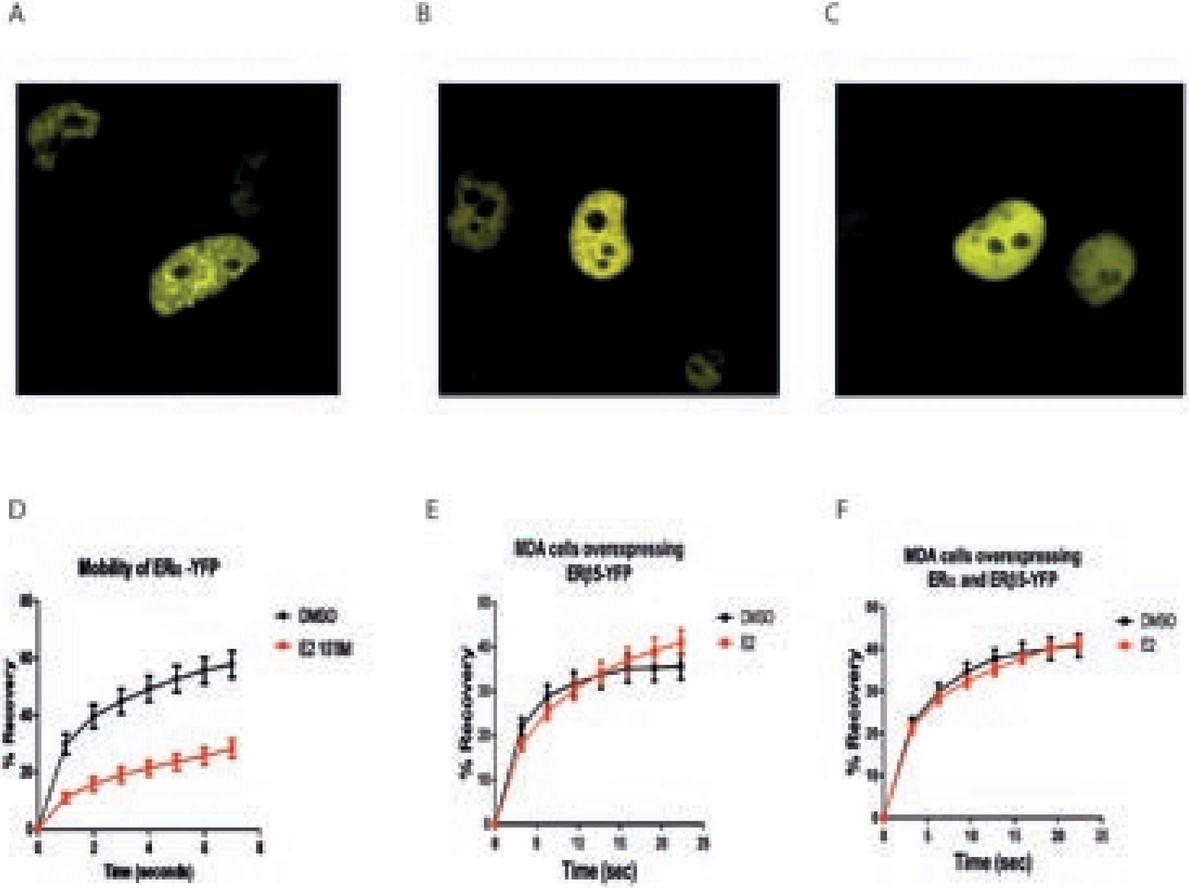
A
B
C
D
E
F
DMSO
E2
% Recovery
Time (seconds)
Time (sec)
MDA cells overexpressing
